# Self-Regulation in Preschool: Examining Its Factor Structure and Associations With Pre-academic Skills and Social-Emotional Competence

**DOI:** 10.3389/fpsyg.2021.717317

**Published:** 2022-01-18

**Authors:** Irem Korucu, Ezgi Ayturk, Jennifer K. Finders, Gina Schnur, Craig S. Bailey, Shauna L. Tominey, Sara A. Schmitt

**Affiliations:** ^1^Child Study Center, Yale University, New Haven, CT, United States; ^2^Department of Medical Social Sciences, Northwestern University, Evanston, IL, United States; ^3^Department of Human Development and Family Studies, Purdue University, West Lafayette, IN, United States; ^4^Extension Family and Community Health Program, Oregon State University, Corvallis, OR, United States

**Keywords:** executive function, behavioral self-regulation, emotion regulation, bifactor model, pre-academic skills, social-emotional competence

## Abstract

Self-regulation in early childhood is an important predictor of success across a variety of indicators in life, including health, well-being, and earnings. Although conceptually self-regulation has been defined as multifaceted, previous research has not investigated whether there is conceptual and empirical overlap between the factors that comprise self-regulation or if they are distinct. In this study, using a bifactor model, we tested the shared and unique variance among self-regulation constructs and prediction to pre-academic and social-emotional skills. The sample included 932 preschool children (*M*_age_ = 48 months, *SD* = 6.55; 49% female), their parents, and their teachers in the United States. Children’s self-regulation was assessed using measures of executive function, behavioral self-regulation, and emotion regulation. The bifactor model demonstrated a common overarching self-regulation factor, as well as distinct executive function and emotion regulation factors. The common overarching self-regulation factor and executive function predicted children’s pre-academic (i.e., mathematics and literacy) and social-emotional skills. The emotion regulation factor predicted children’s social-emotional skills. Identifying the shared and unique aspects of self-regulation may have important implications for supporting children’s regulatory skills as well as their success in school.

## Introduction

Children’s ability to regulate themselves is a key developmental task during early childhood (Allan et al., 2014; Robson et al., 2020). Self-regulation is generally defined as the ability to control thoughts, behaviors, and feelings to achieve goal-directed behaviors and has been conceptualized broadly to include neurological processes [executive function (EF)], EF in overt behavior (behavioral self-regulation), and emotion regulation (McClelland et al., 2018). Despite theoretical perspectives suggesting self-regulation is comprised of EF, behavioral self-regulation, and emotion regulation, there has been little empirical work dedicated to testing how the components of self-regulation remain distinct yet simultaneously comprise an overarching self-regulation factor. Although some studies have examined correlations among some constructs of self-regulation (EF and behavioral self-regulation; McClelland et al., 2014; Finders et al., 2021; EF and emotion regulation; Lieberman, 2007), no studies to date have examined whether these indeed constitute one overarching self-regulation construct while remaining distinct subordinate constructs. Therefore, in this study, we use a bifactor model to explore (1) the extent to which different aspects of children’s self-regulation constitute one overarching self-regulation construct while remaining distinct subordinate constructs, and (2) the extent to which an overall self-regulation construct and/or the individual subordinate constructs predict children’s pre-academic and social-emotional competencies.

### Self-Regulation in Early Childhood

Self-regulation broadly refers to the ability to regulate behavior, cognition, and emotion to pursue goal-directed behaviors (Diamond and Lee, 2011; Hofmann et al., 2012). Self-regulation has received increased attention in various disciplines due to its important role in development across the life span (Posner and Rothbart, 2000; Moffitt et al., 2011; McClelland et al., 2013). In early childhood, self-regulation has been associated with pre-academic skills, including literacy and numeracy, and social-emotional outcomes, including social competence and externalizing and internalizing behaviors (Blair and Razza, 2007; McClelland et al., 2007). In addition, a recent meta-analysis on self-regulation analyzing 150 studies documented that self-regulation in early childhood, measured around age 4, predicted 25 outcomes, including achievement, mental health, and interpersonal behaviors in early and later school years as well as in adulthood (Robson et al., 2020).

Theoretical perspectives suggest that self-regulation is multifaceted and consists of different constructs including EF, behavioral self-regulation, and emotion regulation (Frye et al., 1998; Gottlieb, 2007; Blair et al., 2016; Zelazo et al., 2017). Neurological processes underlying EF involve inhibitory control, the ability to inhibit one behavior in favor of another, cognitive flexibility, the ability to flexibly pay attention, and working memory, the ability to hold and manipulate information in mind and have often been measured using performance-based direct assessments (Zelazo et al., 2013). EF in overt behavior, often called behavioral self-regulation, has also been assessed through performance-based direct assessments (McClelland et al., 2014) as well as teacher and parent-report questionnaires. Emotional self-regulation, defined as the ability to modulate strong emotional reactions with adaptive strategies, has typically been assessed through teacher and parent ratings (Raver, 2004).

The broad definition of self-regulation, as well as the lack of a cohesive framework for defining and measuring self-regulation, has led to different conceptualizations of this construct and its components across various disciplines in developmental (e.g., behavioral self-regulation), cognitive and neuroscience (e.g., EF), and social and personality psychology (e.g., effortful control; see Nigg, 2017 for a review). Although previous research has linked these different components of self-regulation, and multiple calls have been made to integrate them under the broader umbrella term of self-regulation (Zhou et al., 2012; Morrison and Grammer, 2016), no study has empirically tested the theoretical perspective that all comprise a larger self-regulation construct.

### Self-Regulation and Academic Skills

A robust body of literature underscores the importance of self-regulation for children’s concurrent and subsequent school performance (see Robson et al., 2020 for review). The explanation for this association is that children must possess the ability to control their thoughts, feelings, and behaviors in order to navigate complex learning environments (Morrison et al., 2010; Duckworth and Carlson, 2013). Research suggests that broad measures of self-regulation, as well as each of the self-regulation components that are of interest in this study, are related to academic outcomes (Smithers et al., 2018). For instance, findings indicate that children who have stronger EF also tend to have higher academic achievement, particularly in mathematics (Bull et al., 2008; Cragg and Gilmore, 2014; Ahmed et al., 2019). Similarly, results indicate that behavioral self-regulation skills are predictive of growth in mathematics, literacy, and vocabulary during kindergarten (McClelland et al., 2007, 2014; Schmitt et al., 2017; Pandey et al., 2018). Finally, studies have shown that children with increased emotion regulation were found to have higher levels of pre-academic skills and achievement in early childhood (Graziano et al., 2007; Ursache et al., 2012; Kwon et al., 2017; Mattar et al., 2020). What is unclear in this line of work is whether an overarching self-regulation construct or its components are driving the associations between children’s self-regulatory skills and their pre-academic skills.

### Self-Regulation and Social-Emotional Competence

Research has also highlighted relations among self-regulation and social-emotional skills (Rademacher and Koglin, 2019). Children who can regulate attention, behavior, and emotion are thought to better navigate the complex social interactions that frequently necessitate recognizing one’s and others’ emotions and intentions, cooperating with one another, and building relationships (Raver, 2002; McClelland et al., 2007). For instance, EF helps children resist impulsive emotional responses, observe and process the emotions of others, and have more positive social interactions with peers and teachers (Teglasi et al., 2015; Mann et al., 2017; Rademacher and Koglin, 2019). Further, strong behavioral self-regulation supports peer competence and control of positive and negative emotions (Trentacosta and Izard, 2007; Ponitz et al., 2009). Alternatively, poor behavioral self-regulation is linked to increased disruptive and/or aggressive behaviors which can lead to peer rejection and difficulties in forming peer friendships (McClelland et al., 2007). Similarly, research shows that emotion regulation is associated with children’s social-emotional competence, particularly emotion knowledge, which is important for creating successful personal relationships and encourages prosocial responsiveness to peers (Eisenberg et al., 2005; Di Maggio et al., 2016; Ornaghi et al., 2019). Taken together, previous research examining the associations between self-regulation and its components included in the present study demonstrates commonalities in their associations between pre-academic skills and social-emotional competence.

### Prior Factor Analytic Approaches and the Utility of a Bifactor Modeling Approach

Despite the commonalities, extant studies have not assessed the overlap and unique aspects of self-regulation constructs (i.e., EF, behavioral self-regulation, and emotion regulation) and whether there is a unique contribution of each component to pre-academic and social-emotional skills when the shared/common variance is partialed out. Prior factor analytic studies mostly rely on common factor models (i.e., correlated-factor models) or second-order (i.e., hierarchical) factor models when examining multifaceted constructs, and bifactor models have not been utilized to explore self-regulation and its subordinate constructs. The majority of prior work focuses on common factor models, and when a general underlying factor is present, multidimensionally cannot be easily examined in these models. Second-order and bifactor models can account for multidimensionality while acknowledging the presence of a general factor (Reise, 2012), but they differ with regard to how they model the data. In second-order factor models, observed variables are specified to measure first-order factors that represent the components of the general construct, and a higher-order factor accounts for the correlations among the first-order factors. Thus, in second-order factor models, it is assumed that first order factors have direct effects on their indicators, but the second-order factor only has indirect effects on its indicators through the first-order factors. It is also assumed that the second order factor accounts for all the associations between the specific factors. Thus, it is not possible to detect the existence of specific factors (i.e., unique variances of the factors that are not explained by the common higher-order factor) with traditional models (Gustafsson and Balke, 1993).

In contrast to second-order models, bifactor models include general and specific factors, but indicators have two direct effects in these models, one from the general factor and one from the specific factor to which the indicator is assigned. Further, in bifactor models, the general factor and the specific factors are within the same measurement level, and the general factors and specific factors are orthogonal, which allows the model to disentangle the sources of reliable variance in composite and subdomain scores. Thus, bifactor models allow for the examination of the unique effects of the general factor as well as the specific factors, which helps to identify whether self-regulation is indeed an overarching construct for EF, behavioral self-regulation, and emotion regulation. Bifactor models also include prediction to external variables based on specific factors above and beyond the general factor using structural equation models (SEM), which would further our understanding about the associations between self-regulation, its constructs, and pre-academic and social-emotional skills. By employing a bifactor modeling approach, we extend prior work by addressing the need for a better understanding of the empirical structure of self-regulation in early childhood and its association with two important skills, pre-academic and social-emotional skills.

### Present Study

**FIGURE 1:**
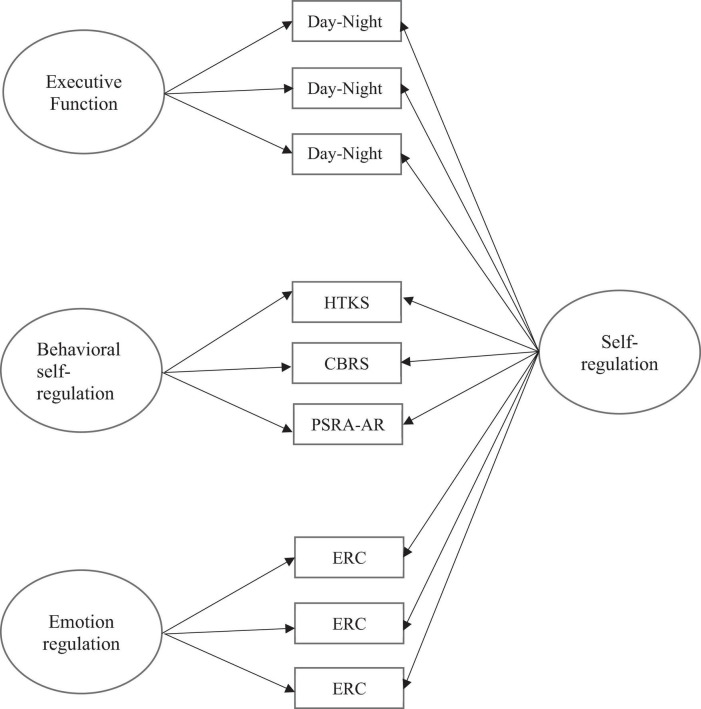
Conceptual bifactor model self-regulation.

In the current study, utilizing a bifactor model, we had two aims: (1) examine the extent to which different aspects of children’s self-regulation constitute one overarching self-regulation construct and the degree to which they are distinct, and (2) explore the extent to which an overarching self-regulation construct (if one emerges from research aim 1) and/or the individual constructs predict children’s pre-academic and social-emotional competencies. We expected that a general overarching self-regulation construct would emerge and predict pre-academic and social-emotional skills. Further, we expected that each construct of self-regulation would also have unique variance and load onto their respective constructs (see Figure 1), and thus, we hypothesized that each would uniquely predict pre-academic and social-emotional skills controlling for child’s age, sex, and family income-to-needs ratio.

## Materials and Methods

### Participants

Participants included 932 preschool-aged children (*M*_age_ = 48 months [*SD* = 6.55]; 49% female), their parents, and their teachers from 62 preschools and 188 classrooms across a Northeastern region of the United States. The sample for this study came from an evaluation of a social-emotional program on children’s school readiness. The sample included racially and ethnically diverse children: 47% White or Caucasian, 28% Black or African American, 11% multiracial, 9% American Indian or Alaskan Native, 4% Asian, and 44% identified as Hispanic or Latinx, representing the broader area in the Northeast region of the United States. Children primarily spoke English (77%), with 14% speaking primarily Spanish, and 9% who were multilingual or spoke other languages.

### Procedures

Data were collected from children, teachers, and parents at one time point in the fall of the preschool year. Participants were recruited from publicly (84%) and privately (5%) funded community-based preschools, 16% of which were Head Start centers serving low-income children and families. Parents of all children at participating schools were sent home an invitation to participate in the study with a letter including a consent form, a description of the study, and a short demographic survey. Trained research assistants directly assessed participating children’s EF, behavioral self-regulation, pre-academic skills, and social-emotional competence after receiving verbal assent from children to participate in the activities. Assessments were conducted in preschools in quiet spaces across two sessions on separate days. Each session took approximately 20 min to complete. For children whose parents indicated that they speak a language other than English, a bilingual research assistant administered a language screening test. Children whose primary language was Spanish and could not pass the screening test in English were assessed in Spanish. Children who spoke a language other than Spanish and did not pass the screening task in English were not administered any of the assessments. Children’s emotion regulation and one aspect of behavioral self-regulation (i.e., Child Behavior Rating Scale) were assessed through teacher reports. After participation in the assessment battery, children received a sticker. Following study participation, teachers received a $30 gift card.

### Measures

#### Executive Function

Children’s EF was measured by the Day-Night Stroop task (Montgomery and Koeltzow, 2010). Research assistants presented cards depicting either a sun or a moon and asked the children to say the opposite of what they saw. For example, children were asked to say “night” when shown a picture of a sun. Children were first given trials to be sure they understood the task and then were given 14 test items. If the child answered correctly, they received a score of 2, similar responses (e.g., “sun” when the correct response is “day”) or a self-correct received a score of 1, and incorrect or no response received a score of 0. This measure has strong reliability and has been shown to be correlated with other EF measures in previous studies (Carlson, 2005). The measure demonstrated high internal consistency in the current sample (Cronbach’s α = 0.93).

#### Behavioral Self-Regulation

Children’s behavioral self-regulation was measured using the Head-Toes-Knees-Shoulders task (HTKS; McClelland et al., 2014), a direct child assessment, the Child Behavior Rating Scale (CBRS; Bronson et al., 1995), a teacher report, and the Preschool Self-Regulation Assessment—Assessor Report (PSRA-AR; Smith-Donald et al., 2007), an assessor report completed by research assistants after administration of direct-child assessments.

##### Head-Toes-Knees-Shoulders

The HTKS measures behavioral self-regulation skills in preschool children, tapping into working memory, inhibitory control, and cognitive flexibility in overt behavior. This direct assessment consists of 30-items across three sections. During the first section of the HTKS, the research assistant asked the child to touch their head and then their toes. Children were then challenged to do the opposite of what the researcher says (e.g., “When I say touch your head, instead of touching your head, touch your toes”). The second section of the HTKS increases in difficulty adding in knees and shoulders (e.g., “When I say touch your knees, instead of touching your knees, you touch your shoulders”). The final section increases in difficulty again by changing the original rules (e.g., “When I say touch your head, instead of touching your head, touch your knees”). Children only continued to the subsequent section if they received four or more points. Correct responses on this task were scored as 2 points, self-correct responses were scored as 1 point, and incorrect responses were scored as 0 points. Scores were summed to create a total score. Previous research with the HTKS has produced strong reliability and validity statistics within diverse samples (McClelland et al., 2014). The HTKS demonstrated high internal consistency in the current sample (Cronbach’s α = 0.93).

##### Child Behavior Rating Scale

The CBRS is a teacher report assessment evaluating a child’s task behavior and social behavior with peers and adults (Bronson et al., 1995). The original measure is comprised of 32 questions, but for the purposes of this study, only the self-regulation subscale was used consisting of 10 questions (e.g., “observes rules and follows directions without reminders”). The classroom self-regulation subscale assesses children’s behavioral self-regulation during academic tasks (e.g., following directions, staying on task), as rated by teachers using a scale from Never (1) to Always (5). A sum score was used in the analyses. The measure has demonstrated high internal consistency in previous studies (Ponitz et al., 2009; Schmitt et al., 2014) and in the current study (Cronbach’s α = 0.95).

##### Preschool Self-Regulation Assessment-Assessor Report

The PSRA-AR is an assessor report of the child’s performance on tasks related to attention/impulse control, compliance, activity level, feelings, and sociability (Smith-Donald et al., 2007). The original assessment is composed of 28 items and adapted from the Leiter-R scale (Roid and Miller, 1997) and Disruptive Behavior-Diagnostic Observation coding system (Wakschlag et al., 2005). The short version of the scale includes 12 questions with two subscales, the attentive/impulse control scale and the positive emotion scale, and provides a global picture of children’s emotion, attention, and behavior throughout the assessor-child interaction during the assessments. Seven items representing the attentive/impulse control subscale were used for the purpose of the current study (e.g., “sustains concentration while doing task; distracted by sounds and sights throughout the assessment”). Assessors rated these items by using a scale from child was not able to concentrate (0) to child was able to concentrate and persist with task (3). A sum score was used in the analyses. The measure has been shown to be reliable and valid in previous studies (Williford et al., 2013; Daneri et al., 2018). The measure demonstrated high internal consistency in the current sample (Cronbach’s α = 0.76).

#### Emotion Regulation

The Emotion Regulation Checklist (ERC; Shields and Cicchetti, 1997) is a teacher-report assessment and measures children’s emotional expression and regulation patterns and skills by items describing situationally appropriate affective displays, empathy, and emotional self-awareness. The emotion regulation subscale of the ERC was used (e.g., “can recover quickly from episodes of upset or distress, does not remain anxious or sad after emotionally distressing events”). It includes 14 items rated on a scale from Never (1) to Almost Always (4). The measure has been shown to be reliable and valid in previous studies (Shields and Cicchetti, 1997; Graziano et al., 2007) and demonstrated high internal consistency in the current sample (Cronbach’s α = 0.74).

#### Pre-academic Skills

The Woodcock Johnson IV Tests of Achievement was used to test children’s literacy and mathematic abilities, specifically the Letter-Word Identification and Applied Problems subtests (Schrank et al., 2014). The Woodcock Johnson Letter-Word Identification (WJLW) subtest assesses children’s developing word-coding skills, including the ability to recognize letters, name letters, and (for children who are advanced on the task) read words. The Woodcock Johnson Applied Problems (WJAP) subtest assesses children’s abilities related to counting objects, reading numbers, and performing basic addition and subtraction. Children are tested until they receive six consecutive questions incorrect for the Letter-Word Identification subtest and five consecutive questions incorrect for the Applied Problems subtest before the research assistant ends the assessment. Correct responses are scored as 1, and incorrect responses are scored as 0. Final scores are calculated by summing correct items for each subtest. The raw scores are then uploaded onto the Woodcock-Johnson Scoring website to obtain the W-scores. W scores are used in the analyses, which are the conversion of raw scores into centered W-scores. The assessment has strong psychometric properties as demonstrated in previous validation studies (McGrew et al., 2014).

#### Social-Emotional Competence

The Affect Knowledge Test (AKT; Denham et al., 2003, 2015; Bassett et al., 2012) was used to evaluate social-emotional competencies. The AKT measures expressive, receptive, and situation emotion knowledge using facial expressions, stereotypical and non-stereotypical vignettes, and a teacher-informed survey. Before children were assessed, teachers completed a short survey of 12 questions asking how the child would normally respond emotionally in various situations, which were then used to inform the presentation of the non-stereotypical vignettes. First, children were presented with felt faces of emotional expressions (i.e., happy, sad, angry, and afraid). Children were asked, “How does she/he feel?” for each emotion to evaluate children’s expressive emotion knowledge. Then, children were asked to “Point to the [emotion] face” for each emotion to assess children’s receptive emotion knowledge. Next, research assistants performed nine vignettes using puppets that depicted children expressing emotions in developmentally appropriate, emotionally charged situations. For the first three vignettes, the puppet depicted the same emotion most children would feel, as an index of children’s stereotypical emotion knowledge. For the remaining six vignettes, the puppet depicted different emotions from what the teacher reported that the child would normally feel (e.g., happy or sad to come to preschool), as an index of children’s non-stereotypical emotion knowledge. Correct responses were given 2 points, responses with the same emotional valence were given 1 point, and incorrect responses were given 0 points. Scores were created for each component (e.g., expressive, receptive, situation emotion knowledge), z-scored, and summed into a total score (Cronbach’s α = 0.87). The AKT has been shown to be reliable and valid in previous studies (Denham et al., 2003, 2015; Bassett et al., 2012).

### Analytical Strategies

The confirmatory bifactor models and the SEM were estimated using the lavaan package (Rosseel, 2012) in R (version = 3.6.2; R Core Team, 2019). Full information maximum likelihood estimation (Rhemtulla et al., 2012) with cluster-robust standard errors and a Satorra-Bentler scaled test statistic were used. The cluster-robust SEs we report were adjusted accounting for the nested structure of the dataset (i.e., children nested within classrooms). We first examined an *a priori* measurement model: a bifactor model in which a single general factor accounts for the shared variance among the indicators of the EF, behavioral self-regulation, and emotion regulation, and three orthogonal specific factors representing EF, behavioral self-regulation, and emotion regulation that account for the remaining common variance among their respective indicators. Since EF and emotion regulation were measured using single measures, we generated three random parcels for each to make sure we had an equal number of indicators in each domain-specific factor. In this configuration, EF was measured with three random parcels of the Day-Night Stroop task, behavioral self-regulation was measured with CBRS, HTKS, and PSRA scores, and emotion regulation was measured with three random parcels of the ERC item scores. The latent scales (i.e., both the specific and general) of the bifactor model were defined by fixing the variance of the latent factors to one.

We used a range of goodness-of-fit indices for model evaluation. The χ^2^ statistic compares the observed and the model-implied covariance matrices. A non-significant χ^2^-test indicates a close correspondence between the model and the sample data. However, as widely acknowledged, Type I error rates of the χ^2^-test inflate with increased sample sizes (>200; Steiger, 2007; Van de Schoot et al., 2012). The Comparative Fit Index (CFI; Bentler, 1990) assesses how much better the specified model fits the sample data compared to a baseline model in which the observed variables are uncorrelated. CFI values ≥ 0.90 and ideally ≥ 0.93 indicate adequate fit (Byrne, 1994; Hu and Bentler, 1999). The root mean square error of approximation (RMSEA; Steiger and Lind, 1980) represents the discrepancy between the model and observed covariances per degree of freedom and can be considered a measure of effect size. We considered point estimates of the RMSEA values < 0.08 as an indication of acceptable fit. We also expected a good fitting model to produce an upper bound of the 90% confidence-interval of the RMSEA < 0.10 (Browne and Cudeck, 1992). Mardia’s (1970) multivariate skewness (b-1*p*. = 7.43, skewness = 771.90, *p* < 0.001) and kurtosis (b-2*p*. = 99.79, kurtosis = 0.71, *p* = 0.43) tests indicated multivariate non-normality of the data; therefore, we reported the robust versions of these fit indices (univariate descriptive statistics are presented in Table 1). In addition to the goodness-of-fit indices, we evaluated the pattern of factor loadings to examine the relative strength of the relations between the observed variables and the general factor and their respective domain-specific factors.

**TABLE 1:** Descriptive statistics and correlations for study variables.

	Day-Night	CBRS	PSRA-AR	HTKS	ERC	Age	Sex	Income-to-needs	WJ-LW	WJ-AP	AKT
Day-Night	−										
CBRS	0.22***	−									
PSRA-AR	0.04	–0.03	−								
HTKS	0.32***	0.19***	–0.03	−							
ERC	0.17***	0.55***	–0.04	0.12**	−						
Age	0.20***	0.19***	–0.12***	0.26**	0.10**	−					
Sex	0.03	0.24***	0.00	0.05	0.12**	–0.03	−				
Income-to needs	0.12**	0.12***	0.03	0.12**	0.13**	–0.05	0.01	−			
WJ-LW	0.25***	0.25**	–0.02	0.31***	0.16***	0.31***	0.06	0.22***	−		
WJ-AP	0.37***	0.36***	−0.07*	0.40***	0.23***	0.39***	0.05	0.29***	0.51***	−	
AKT	0.21***	0.19***	0.19***	0.14***	0.18***	0.29***	–0.02	0.13**	0.33***	0.55***	−
N	783	849	922	793	832	932	885	630	828	828	806
Mean	15.07	33.98	9.35	6.40	21.86	47.89	0.49	3.55	322.04	384.1	0
SD	9.72	8.22	1.87	10.44	3.61	6.55	−	4.83	28.49	27.41	4.16
Minimum	0	10	3	0	9	36.01	−	0	226	324	–13.4
Maximum	28	50	21	52	28	67.61	−	58.25	478	453	3.79
Skewness	–0.19	–0.11	2.3	1.97	–0.49	–0.05	−	4.36	0.27	–0.32	–1.36
Kurtosis	–1.29	–0.45	11.5	3.42	–0.12	–1.06	−	32.07	1.67	–0.38	1.19

*p < 0.05, **p < 0.01, ***p < 0.001.

Next, we ran a series of SEMs to investigate the unique relations of the general and specific factors of the bifactor model of self-regulation with the scores from three outcome measures of pre-academic skills (WJLW and WJAP) and social-emotional competence (AKT). In these analyses, we controlled for several demographic characteristics, including children’s age, sex, and family income-to-needs ratio (calculated by dividing the participant reported annual family income by the federal poverty level in 2019).

## Results

### Measurement Model

**FIGURE 2:**
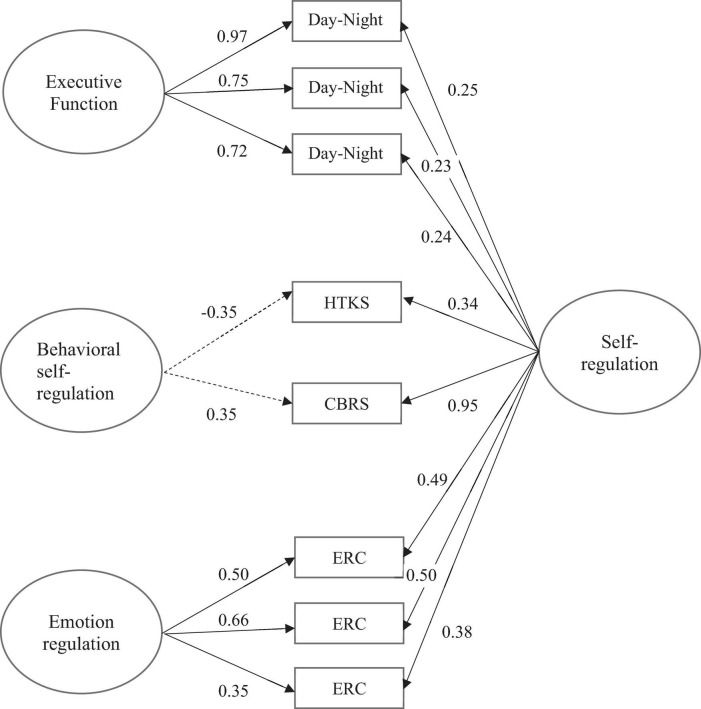
Tested bifactor model self-regulation.

The bifactor model with one general and three specific factors (i.e., EF, behavioral self-regulation, emotion regulation), each of which were measured by three indicators, produced acceptable model fit, χ^2^(18) = 77.00, *p* < 0.001, RMSEA = 0.059, RMSEA 95% CI = [0.046, 0.073], CFI = 0.976. Next, we examined the factor loadings. All indicators except for the assessor report measure of the behavioral self-regulation (PSRA-AR) showed positive and statistically significant factor loadings to the general factor, providing support for the existence of an overarching factor. Since the PRSA-AR did not load significantly on the general factor (standardized factor loading = –0.047, *SE* = 0.071, *p* = 0.503, 95% CI = [–0.186, 0.091]), we removed this measure from the model and refit another bifactor model where behavioral self-regulation was measured with the remaining two indicators (i.e., CBRS and HTKS). This model produced good model fit, χ^2^(12) = 61.59, *p* < 0.001, RMSEA = 0.068, RMSEA 95% CI = [0.052, 0.086], CFI = 0.979. All indicators showed significant and positive loadings on the general factor, *p*s < 0.001, thus, we continued with this model (see Figure 2). Standardized factor loadings for the general factor ranged from 0.20 to 0.95 and are presented in Table 2.

**TABLE 2:** Standardized factor loadings from the bifactor model.

	General	EF	Behavioral self-regulation	Emotion regulation
Day-Night parcel 1	0.25 [0.05]	0.97 [0.01]		
Day-Night parcel 2	0.23 [0.05]	0.75 [0.02]		
Day-Night parcel 3	0.24 [0.05]	0.72 [0.02]		
CBRS self-regulation	0.95 [0.12]		0.35 [1.02]^a^	
HTKS	0.34 [0.09]		–0.35 [1.31]^a^	
ERC-parcel 1	0.49 [0.07]			0.50 [0.08]
ERC-parcel 2	0.50 [0.07]			0.66 [0.07]
ERC-parcel 3	0.38 [0.06]			0.35 [0.07]

*^a^p-value > 0.05; Rest of the loadings were significant at α = 0.001.*

Controlling for the general factor, domain-specific factor loadings for the EF and emotion regulation factors were positive and statistically significant, *p* < 0.001. However, domain-specific factor loadings of the indicators of the behavioral self-regulation did not reach statistical significance indicating that the systematic variance in these measures was completely accounted for by the general factor.

Relative strength of the loadings to the general and specific factors for a given indicator informs how strongly the indicator measures the general and the respective domain-specific factor. The indicators of EF showed higher loadings to the domain-specific factor (0.72–0.97) than the general factor (0.23–0.25), implying that they are better measures of the domain-specific factor. Indicators of behavioral self-regulation showed statistically significant loadings only onto the general factor (0.34–0.95), implying that they are better measures of the general overarching self-regulation factor. Indicators of the emotion regulation factor showed comparable loadings to the general factor (0.38–0.49) and the specific factor (0.35–0.66), suggesting that they are equally good measures of the general and the domain-specific factors.

Next, we used this bifactor model to examine the unique relations of the domain-specific factors and the general factor with children‘s pre-academic and social-emotional competencies controlling for age, sex, and family income-to-needs ratio. Since behavioral self-regulation did not show significant loadings to the domain-specific factor once the general factor was controlled for, we excluded it from the prediction analyses. Specifically, we examined the degree to which the general factor that emerged in the bifactor model, as well as the specific factors, EF and emotion regulation, were related to pre-academic skills and social-emotional competence when controlling for the general factor.

### Structural Equation Models

#### Woodcock Johnson Letter-Word Identification

The model fit of the SEM regressing WJLW scores on the general factor and EF and emotion regulation specific factors in the bifactor model controlling for children’s age, sex, and income-to-needs ratio was acceptable, χ^2^(41) = 274.40, *p* < 0.001, RMSEA = 0.080, CFI = 0.91. The general factor significantly predicted WJLW scores, β = 0.24, 95% CI = [0.14, 0.33], *SE* = 0.05, *z*-value = 4.95, *p* < 0.001. Additionally, controlling for the covariates and the general factor, the EF domain-specific factor was also positively and significantly related to WJLW scores, β = 0.07, 95% CI = [0.02, 0.13], *SE* = 0.03, *z*-value = 2.54, *p* = 0.012. However, the remaining systematic variance in the emotion regulation specific factor was not significantly associated with WJLW scores, β = –0.01, 95% CI = [–0.12, 0.10], *SE* = 0.06, *z*-value = –0.10, *p* = 0.923. Among the covariates, age and income-to-needs ratio were significantly related to WJLW scores, whereas sex was not (see Table 3).

**TABLE 3:** Structural equation models between self-regulation factors and academic and social-emotional skills.

	WJLW			WJAP			AKT		
	β	*SE*	*b*	β	*SE*	*b*	β	*SE*	*b*
Age	0.26***	0.03	1.12	0.30***	0.04	1.23	0.25***	0.04	1.16
Sex	0.02	0.03	0.87	–0.03	0.03	–1.39	–0.04	0.04	–0.30
Income-to-needs	0.25***	0.05	1.43	0.28***	0.04	1.51	0.12*	0.05	0.10
General self-regulation factor	0.24***	0.05	6.56	0.42***	0.06	11.03	0.15***	0.04	0.62
EF	0.07*	0.03	2.02	0.11**	0.03	3.00	0.10**	0.03	0.39
Emotion regulation	–0.01	0.06	–0.15	–0.11	0.10	–2.92	0.10*	0.05	0.43

*p < 0.05, **p < 0.01, ***p < 0.001. The structural estimates for the EF and emotion regulation reflect residual estimates after accounting for the general self-regulation factor.

#### Woodcock Johnson Applied Problems

The model fit when regressing WJAP scores onto the general and domain-specific factors, controlling for the same covariates, was acceptable, χ^2^(41) = 292.40, *p* < 0.001, RMSEA = 0.081, CFI = 0.91. The general factor significantly predicted WJAP scores, = 0.42, 95% CI = [0.30, 0.54], *SE* = 0.06, *z*-value = 6.90, *p* < 0.001. Additionally, controlling for the covariates and the general factor, the EF domain-specific factor was also positively and significantly related to WJAP scores, β = 0.11, 95% CI = [0.05, 0.18], *SE* = 0.03, *z*-value = 3.31, *p* < 0.001. However, the remaining systematic variance in the emotion regulation specific factor was not significantly associated with WJAP scores, β = –0.11, 95% CI = [–0.30, 0.08], *SE* = 0.10, *z*-value = –1.14, *p* = 0.253. Among the covariates, age and income-to-needs were significantly related to WJAP scores, whereas sex was not (see Table 3).

#### Affect Knowledge Test

Regressing the AKT scores on the general factor and the EF and emotion regulation domain-specific factors in the bifactor model, controlling for the covariates, produced acceptable fit, χ^2^(41) = 261.21, *p* < 0.001, RMSEA = 0.078, CFI = 0.92. Controlling for the covariates, the general factor significantly predicted AKT scores, β = 0.15, 95% CI = [0.07, 0.24], *SE* = 0.04, *z*-value = 3.52, *p* < 0.001. Controlling for the covariates and the general factor, both the EF, β = 0.10, 95% CI = [0.04, 0.16], *SE* = 0.03, *z*-value = 3.10, *p* = 0.002, and emotion regulation, β = 0.10, 95% CI = [0.01, 0.20], *SE* = 0.05, *z*-value = 2.10, *p* = 0.037 specific factors were positively and significantly related to AKT scores. Among the covariates, age and income-to-needs were significantly related to AKT scores, whereas sex was not (see Table 3).

## Discussion

This study examined the extent to which different aspects of self-regulation constitute one overarching self-regulation construct while partialing out the degree to which the aspects were distinct. This study also examined the extent to which an overall self-regulation factor and its individual constructs were associated with children’s pre-academic and social-emotional competencies. Using a bifactor model, the analysis revealed an overarching self-regulation construct, and both the general self-regulation construct and the EF and emotion regulation specific constructs, were differentially related to pre-academic and social-emotional competencies, even after partialing out the general self-regulation construct. This study provides empirical support for theoretical models indicating that self-regulation is indeed a multifaceted construct that also encompasses multiple factors. A better understanding of the structural framework of self-regulation and how its constructs can be aggregated or disaggregated helps the field synthesize various ways of referencing the self-regulation construct. Our study also documented that the self-regulation constructs differentially relate to children’s outcomes, which can set the stage for better supporting certain outcomes through a broader overarching self-regulation construct or its specific factors.

### Self-Regulation as an Overarching and Multifaceted Construct

Self-regulation has been identified as a pivotal skill in early childhood due to its malleability and importance for various short- and long-term outcomes (Diamond, 2002). In line with the conceptualization of self-regulation, three of its constructs, namely EF, behavioral self-regulation, and emotion regulation, were tested in a bifactor model. Results showed that these three constructs of self-regulation significantly contributed to an overarching self-regulation factor. This is in line with the conceptual definition of self-regulation and supports the notion that there are common conceptual underpinnings of each of these self-regulation constructs (Diamond and Lee, 2011; Hofmann et al., 2012; McClelland et al., 2018). Despite the vast amount of interest in this construct, a lack of conceptual clarity across various disciplines, as well as debate over its underlying constructs, make it challenging to study, measure, and support these skills in early childhood (McClelland and Cameron, 2011; Morrison and Grammer, 2016). Our results contribute to this discussion by providing empirical support that the self-regulation construct is multifaceted and there is a common variance shared by its constructs.

Our results indicate that there is substantial shared variance across the three constructs of self-regulation. Although it is unknown what the common variance shared by all the constructs of self-regulation is, it could be attentional processes underlying self-regulated action. In fact, the executive attention network has been proposed to underlie the development of conscious control and be responsible for monitoring and resolving conflict between other brain networks (Rothbart et al., 2007). Within this model, executive attention is defined as a limited capacity attentional resource underlying the goal-directed control of cognition, behavior, and emotion (Rueda et al., 2012). Recent evidence has shown that executive attention is the common cognitive factor underlying the self-regulatory capacities captured by EF and effortful control (Tiego et al., 2018). Executive attention is also related to emotion regulation in children (Sheese et al., 2008; McClelland et al., 2015) and is considered to be a mechanism underlying the ability to regulate emotion in order to behave in socially acceptable ways (Eisenberg et al., 2004). Children use various strategies to engage in emotion regulation, including distractive or cognitive strategies that involve redirecting attention or reframing the situation, and these rely on attention (Zimmermann and Stansbury, 2003; McClelland et al., 2018). Thus, it is possible that executive attention and attentional control may be a common process among the various aspects of self-regulation.

It is also plausible that the general self-regulation factor is picking up on the behavioral self-regulation skills captured in the direct assessments and adult reports across the constructs. Indeed, results indicated that the behavioral self-regulation construct completely loaded onto the general self-regulation factor. Behavioral self-regulation is used while integrating and applying EF skills and emotion regulation in a variety of contexts (McClelland et al., 2007). For instance, children need to remember instructions, stop an action to do another action, and flexibly switch between competing rules to complete EF tasks. Similarly, in a classroom setting, raising your hand before talking, switching from play to a clean-up activity, and waiting your turn before participating in a group setting necessitates using behavioral-self regulation skills for both top-down and bottom-up regulation of thoughts and feelings. Thus, it is a reasonable hypothesis that the general self-regulation factor may be picking up on the behavioral self-regulation skills that are represented across all tasks and measures in the current study; however, it will be important for future studies to attempt to identify this factor more precisely.

### Individual Constructs of Self-Regulation

After accounting for the general overarching self-regulation construct, our results showed that the EF and emotion regulation constructs significantly loaded onto their respective factors, but behavioral self-regulation did not load onto its respective construct. These results mean that the systematic variance of behavioral self-regulation measures was fully accounted for by the general self-regulation factor. However, systematic variance in the EF and emotion regulation constructs remained even after accounting for the overarching self-regulation construct.

The remaining variance of EF, after accounting for the overarching self-regulation construct, may reflect the regulation of attention, memory, or thoughts in the absence of overt behavior or salient emotion. Although EF and self-regulation terms are sometimes used interchangeably (Zelazo and Cunningham, 2007), EF is used for purposes other than self-regulation and should not be simply equated with self-regulation (Nigg, 2017). For instance, solving a mental math problem requires EF, as documented in many studies showing a strong association between EF and math (Clements et al., 2016; Schmitt et al., 2017). EF in this context is likely purely cognitive as solving a mental math problem typically doesn’t require behavioral or emotional control.

The other construct with remaining unique variance in the bifactor model after accounting for variance explained by the general factor was emotion regulation. Though emotion regulation has been studied as a complete area of itself, it has also been studied in relation to or as a component of self-regulation (Eisenberg et al., 2004; Zelazo and Cunningham, 2007; Blair et al., 2016). In the current study, after the common variance between cognitive, behavioral, and emotional self-regulation is accounted for, what is left in the emotion regulation construct may be emotion-related processes. Emotion regulation involves attempts to influence which emotions a child has, when the child has them, and how the child experiences and expresses these emotions (Gross, 2015). Thus, the unique variance associated with the emotion regulation aspect of self-regulation may be the processes used to manage both the frequency and the intensity of emotions, emotion related physiological states, and intrinsic regulation of emotions (Eisenberg et al., 2007). In fact, the measure we used to assess emotion regulation was a teacher report that included items tapping into emotion related process such as “can say when feeling sad, angry or mad, fearful or afraid” and “is a cheerful child”, in addition to items tapping into emotion regulation such as “does not remain anxious or sad after emotionally distressing events”.

### Predictions to Pre-academic Skills and Social-Emotional Competence

#### Overarching Self-Regulation Construct

Although it is not clear what the shared variance across the self-regulation constructs exactly represents, our results indicate that the general overarching construct of self-regulation predicts pre-academic (i.e., mathematics and literacy) and social-emotional skills, which is in line with extant literature (Blair and Razza, 2007; Robson et al., 2020). Our results provide new empirical evidence that all the different constructs of self-regulation, including cognitive, behavioral, and emotional, share a common process, and this common process is positively and significantly associated with pre-academic and social-emotional skills controlling for children’s age, sex, and family income-to-needs ratio.

#### Executive Function

Even though empirical research exploring the overall and unique contributions of the cognitive, behavioral, and emotional self-regulation constructs at the same time is scarce, our findings support previous research examining the unique effects of EF and behavioral self-regulation on pre-academic skills. When examined simultaneously, EF contributed to children’s academic skills whereas behavioral self-regulation (called classroom self-regulation) did not (Morgan et al., 2018; Finders et al., 2021). Our findings expand previous work by parsing out the common variance in the constructs of self-regulation and by exploring the unique predictive abilities of these constructs in a bifactor modeling approach. Our bifactor results showed that the remaining variance in EF is still significantly associated with pre-academic skills, including math and literacy, as well as social-emotional skills, even after its shared variance with the general overarching self-regulation construct has been accounted for. This is consistent with prior research that demonstrates relations between EF and pre-academic skills (McClelland et al., 2007; Morrison et al., 2010; Allan et al., 2014; Schmitt et al., 2017), as well as social-emotional competence (Riggs et al., 2006; Korucu et al., 2017).

#### Emotion Regulation

Even though emotion regulation has been conceptually linked to children’s pre-academic skills, little research has empirically tested this association (Sanson et al., 2004; Raver et al., 2007; Ursache et al., 2012). In contrast, prior evidence of relations between emotion regulation and social-emotional skills has been well-documented (Eisenberg et al., 2005; Di Maggio et al., 2016). Our bifactor results showed that after accounting for the common variance in the constructs of self-regulation, the unique variance of emotion regulation was positively and significantly associated with children’s social-emotional competencies, but not with pre-academic skills. This is in line with studies documenting positive and significant associations between emotion regulation and social-emotional competence (Housman et al., 2018: Ornaghi et al., 2019). The non-significant finding between emotion regulation and pre-academic skills in this study is also in line with prior work showing only indirect associations between the two (Howse et al., 2003). For instance, it is argued that emotion regulation may be associated with academic skills through other factors such as teacher-child relationships and motivation (Graziano et al., 2007). Thus, there is need for more research to further disentangle the associations and mechanisms between emotion regulation and pre-academic skills.

### Limitations and Future Directions

The current study contributes to our understanding of the common and unique aspects of self-regulation constructs and has several strengths. We utilized a large, diverse sample, and a statistical approach that enabled us to disentangle the sources of systematic variance in the domain-specific and general factors of the self-regulation construct. Using a bifactor model allowed us to examine the common and unique aspects of self-regulation constructs at the same time and their overall or unique associations with two important skills: pre-academic and social-emotional skills. Using a bifactor model overcame measurement challenges of total vs. individual score approaches by allowing us to consider measurement error in the models. Employing a bifactor model also helped us to overcome challenges with the reflective latent scoring approach, which assumes unique variance that is not common among the constructs is a measurement error. Finally, using a bifactor model overcame challenges with the second order models and allowed us to examine unique relations between the domain-specific factors with outcome variables above and beyond the general factor using standard SEM (since they are represented as latent factors as opposed to disturbances in the model; Chen et al., 2006; Reise, 2012).

Despite its strengths, the current study does have several limitations. First, even though we used multiple methods to assess various self-regulation constructs, including direct assessments, a teacher-report assessment, and an assessor report (observation during task administration), this only applies to the behavioral self-regulation construct, providing a deeper and more nuanced set of measurements for this construct than the others. The other self-regulation constructs, EF and emotion regulation, were each only assessed with one measure. Specifically, the task we used for assessing EF, the Day-Night task, taps primarily into inhibitory control and working memory. Future research should use multiple measures to assess EF and its components (e.g., cognitive flexibility) and emotion regulation as measure selection may have influenced our findings. Including multiple types of measurement for each of these constructs in future studies could strengthen the current study findings or suggest alternate relations between the distinct and overarching self-regulation constructs.

Further, task impurity problems in the field of self-regulation and EF may influence the conclusions that could be drawn from this study. Even though the measures used in the study have been found to reliably represent their constructs in previous studies, measures (e.g., HTKS) used to assess EF and behavioral self-regulation constructs have often been used interchangeably. Further, measures used to represent behavioral-self regulation could include aspects of emotion regulation depending on the context in which it is being evaluated. For instance, teachers’ rating of classroom self-regulation, considered as a behavioral self-regulation measure in the current study, may include ratings of subtle emotion regulation strategies (e.g., “attempts new challenging tasks”). Similarly, the task that has been used to represent EF in this study may have common components with the task that assesses behavioral self-regulation (e.g., inhibitory control). Thus, and as with any study, the findings observed may be an artifact of the measures used in this study, and it will be important for future research to replicate findings to determine whether the overall or unique associations of the self-regulation constructs and their associations with pre-academic and social-emotional skills will hold when different sets of measures are used.

Though this is a problem with any cognitive assessment in the field, it is difficult to identify what is common and shared between the various self-regulation constructs. What is shared among the self-regulation constructs used in the study might reflect another, non-cognitive, and non-measured ability (e.g., motivation) especially among the direct assessments. Thus, our conceptualization of a general overarching self-regulation construct may include such factors. Relatedly, not all of the potential self-regulation constructs were tested in the current study. Delay of gratification and effortful control could also be tested under the self-regulation construct (McClelland et al., 2018), though task impurity may be a problem here as well.

Another limitation of the current study is that the data were collected at a single time point and thus the results are cross-sectional limiting application of results to change in self-regulation over time. It will be important for future research to replicate the findings with longitudinal data and to investigate the common and unique aspects of self-regulation constructs at different time points, as well as the unique and overlapping predictive abilities of these constructs with academic outcomes over time. Further, it is important to emphasize that the PSRA-AR assessment did not load significantly on the general overarching self-regulation factor, and thus was removed from the measurement model. Future research should replicate these findings and also consider using other measures to observe children’s behavioral self-regulation performance during task administration. Although the current study included racially and ethnically diverse children, results may not generalize to populations outside of the U.S. Finally, it is also important to note that although our methodology, the bifactor model, overcame challenges with the reflective scoring approach (by treating the unique variances as orthogonal factors as opposed to measurement error), there is an ongoing debate about using the reflective vs. formative scoring approach specifically with regards to EF and its components (Willoughby, 2014; Camerota et al., 2020). Future research may benefit from examining these different approaches in alignment with research questions.

## Conclusion

Research suggests that self-regulation is an important predictor of various outcomes in early childhood and beyond, including academic achievement, social-emotional skills, and health and other life outcomes, including earnings and criminal charges (Moffitt et al., 2011; Robson et al., 2020). Although it is acknowledged that self-regulation is a multifaceted construct, it is unclear how its constructs that tap into cognitive, behavioral, and emotional aspects are connected and whether they constitute an overarching self-regulation construct. This study, to our knowledge, was the first to use a bifactor model to explore these relations. Findings indicated that self-regulation is indeed a multifaceted construct, yet the EF and emotion regulation constructs of self-regulation also have unique variances. Further, these constructs differentially predicted pre-academic and social-emotional skills, such that the overarching self-regulation factor and specific EF factor both predicted academic and social-emotional skills, and the emotion regulation factor predicted social-emotional skills. Study findings may have important implications for supporting children’s regulatory skills as well as their success in school. Specifically, identifying specific aspects of self-regulation most predictive of early academic achievement and social-emotional skills can help early childhood programs strategically and intentionally support targeted skill development, putting time and resources into fostering the most beneficial skills during the early childhood years.

## Data Availability Statement

The raw data supporting the conclusions of this article will be made available by the authors, when the larger study is completed. The study is still ongoing. Requests to access the data should be directed to IK, irem.korucu@yale.edu.

## Ethics Statement

The studies involving human participants were reviewed and approved by the Yale University Institutional Review Board. Written informed consent to participate in this study was provided by the participants’ legal guardian/next of kin.

## Author Contributions

IK, ST, and SS contributed to the conceptualization and design of the study. IK and EA contributed to the data analysis, results, and writing. JF, GS, and all others contributed to the writing and editing of the manuscript. All authors contributed to the article and approved the submitted version.

## Conflict of Interest

The authors declare that the research was conducted in the absence of any commercial or financial relationships that could be construed as a potential conflict of interest.

## Publisher’s Note

All claims expressed in this article are solely those of the authors and do not necessarily represent those of their affiliated organizations, or those of the publisher, the editors and the reviewers. Any product that may be evaluated in this article, or claim that may be made by its manufacturer, is not guaranteed or endorsed by the publisher.
